# Medical Society of London

**Published:** 1845-03

**Authors:** 


### RECORD OF MEDICAL SCIENCE.

### MEDICAL SOCIETY OF LONDON, MONDAY, DECEMBER 2, 1844.

Mr. Dendy read a letter on the *Tolerance of Disease.*

He said, that during our pathological investigations we were very often surprised, and sometimes astonished, at the extensive organic lesions and encroachments which took place in the body with so little comparative distress, or even indication, during life. This was, of course, often dependent on the natural powers of resistance to disease, but chiefly on those which were the result of a slow or gradual development of the morbid condition.

In the double organs, especially those of the brain, lungs, and kidneys, this fact was often beautifully evident. A cerebral hemisphere might be reduced to a pulp, a lung hepatized, or a kidney absorbed, and yet mind, and respiration, and urinary secretion might go on without any very extraordinary interruption or derangement.

Some years ago he related to the society the case of a young lady of Wandsworth, in whose brain there was a large tubercle, involving the corpora striata and the thalami, and a ventricle full of yellow serum, and yet, although severe symptoms had been present, the child was occasionally cheerful and smiling, and had apparently even enjoyed herself a day or two before her death.

*Case of Hobnailed Liver.*—Two other cases had lately been under his care, which again illustrated this subject.

One of these was the case of a lady, residing in Piccadilly, of about forty years of age, whom he had himself attended for various maladies during the last three years. She had, however, during almost her whole life, been afflicted by many severe ailments, and yet through all this had preserved a cheerful and serene disposition. When about twenty years old, she was the subject of a series of chronic abscesses, during the course of which she was reduced to a skeleton, her arm having been twice sentenced to amputation. The limb was, however, preserved. About a year ago she suffered under severe and complicated hepatic derangement, attended by very acute pain and sleepless nights, this being, indeed, the third attack she had experienced. On examining the abdomen, he was surprised to find it distended irregularly, the liver, especially, being extremely tumid and indurated; and through the integuments it was clearly ascertained that the viscus was extensively diseased, with a nobulated deposit, by some, he believed, termed the "hobnail liver." This condition, she assured him, she had laboured under for many years. On her convalescence from the acute stage of this condition, he sent her to Essex, and on her return, although she seemed happy and had gained flesh, he found a large, red, angry swelling on her back. This variety of anthrax he attempted to subdue, but it sloughed extensively; and, as it adhered firmly for some time, he dissected the greater portion from its attachments. This was now nearly well, and the lady herself apparently so, but she still bore about with her a studded and tumefied liver.

*Extensive Scrofulous Disease.*—The other case was that of a child, eight years old, an infirmary patient. About a year ago she was presented to him with the ends of her fingers and thumbs enlarged and discoloured, with other marks of struma. Iodine, internally, reduced the swelling of the fingers very nearly to their natural size, and he had prepared the case as one illustrative of the beneficial influence of iodine in struma.

About a month since she was again admitted, complaining of lumbar pains, &c., the secretion of urine being very scanty, passing almost guttatim. Her sister at this time lying ill with variola, Mr. Ainger, saw her at home a day or two after, and although there was no prominent symptom to fix on, he suspected she might be labouring under suppressed variola. On the next day, without exciting much previous attention or anxiety among her friends, she suddenly died, without convulsion or other evident cause of dissolution.

In about thirty hours, Dr. Willshire, Mr. Ainger, and himself examined the body, and the appearances he would thus briefly describe:—

Alimentary canal and liver healthy. Heart flabby, with some yellow specks on its surface. Lungs partially hepatized, and also studded with tubercles of various sizes, but not suppurating. Spleen very much enlarged and gorged, of a deep crimson hue, also studded thickly with large tubercular spots; its weight one pound. The left kidney nearly three times its normal size, also tuberculated; the right not much enlarged, but indurated, nodulated, and misshapen. The thyroid and cervical glands were extensively diseased, some indurated, others softer, and tuberculated; and they were so firmly pressing on the cervical and air-passages, that it seemed wonderful that cerebral circulation or respiration could, even for an hour, be carried on, and yet neither delirium nor convulsive action, nor dyspnœa were at any time observed during her illness.

Several cases showing the tolerance of disease were related.

The President mentioned a case in which the spinal cord, at one portion, was so soft, through its entire extent, that it could be poured from its canal: the only symptom had been constipation.

Mr. A. Fisher detailed a case of abscess, as large as a hen's egg, filling up nearly one hemisphere of the cerebellum, in which the only symptom preceding death was a violent earache, which continued for twenty-four hours.

Mr. Headland related a case of extensive suppuration, affecting the white matter of both hemispheres of the brain, but more particularly the left, the suppuration extending on that side to the lateral ventricle. There were no previous symptoms, except within a day or two of her death, when she was suddenly seized with most violent pain in the head, succeeded by rigors, intolerance of light, and a remarkably rapid pulse. The intellectual faculties remained perfect to the last. He related a case, also, in which a boy lost a portion of his brain from a kick by a horse, and recovered.

Mr. Stedman alluded to the case of a man who was stabbed by a bullock, the horn passing through one of his eyes and out at the upper part of the head. He lived forty-eight hours ; intellect was perfect to the last. He related a case, also, of atrophy of one entire lung, in which the patient never complained, and pursued his work of threshing till a very short time before death.

Dr. Leonard Stewart recollected seeing a brain studded all over with tubercles, in which no symptom had existed during life.

Dr. Garrod had seen a case in University College Hospital, of a woman who complained only of general *malaise*, slight cough and dyspnœa, with occasional pain in the back. Every organ was found after death, to be affected with cancer.—*London Lancet.*

